# Supplementary material for: Drep-2 is a novel synaptic protein important for learning and memory
Source: eLife. 2014 Nov 13;3:e03895. doi: 10.7554/eLife.03895 (PMC4229683; doi:10.7554/eLife.03895)
Supplement: Supplementary file 1. — Mass spectrometry: core proteins enriched over both controls. The table shows proteins enriched at an FDR of 1%. Ranks are based on the ratios of Drep-2GFP IPs, GFP beads vs. plain beads (compare Figure 7A–B). Putative functions were derived from Flybase. GFP (#6) was removed from this list. DOI: http://dx.doi.org/10.7554/eLife.03895.015 [file elife03895s001.docx]

| **Rank** | **Flybase name** | **Name in network** | **Full name** | **CG number** | **Flybase ID** | **Network class I** | **Network class II** | **Flybase: molecular function** |
| --- | --- | --- | --- | --- | --- | --- | --- | --- |
| 1 | crb | Crumbs | Crumbs | CG6383 | FBgn0259685 | Membrane | Cytoskeleton | protein kinase C binding |
| 2 | cos | Cos2 | Costal-2 | CG1708 | FBgn0000352 | Cytoskeleton | Membrane | smoothened binding; microtubule binding; protein binding;  protein kinase binding; transcription factor binding |
| 3 | sif | SIF | Still life | CG34418 | FBgn0085447 | G-protein | Membrane | Rac guanyl-nucleotide exchange factor activity |
| 4 | Capr | Caprin | Caprin | CG18811 | FBgn0042134 | RNA |  | RNA binding |
| 5 | mbo | Nup88 | Members only | CG6819 | FBgn0026207 | Membrane |  | protein binding |
| 7 | Drep-2 | Drep-2 | DNA fragmentation factor-related protein 2 | CG1975 | FBgn0028408 | Drep-2 | Membrane | unknown |
| 8 | CG14095 | CG14095 |  | CG14095 | FBgn0036870 | Unrelated | Cuticle | unknown |
| 9 | RnpS1 | RnpS1 | RNA-binding protein S1 | CG16788 | FBgn0037707 | RNA |  | mRNA binding |
| 10 | CG30122 | hnRNP U | Similar to hnRNP U | CG30122 | FBgn0050122 | RNA |  | mRNA binding |
| 11 | Drep-3 | Drep-3 | DNA fragmentation factor-related protein 3 | CG8364 | FBgn0028407 | Membrane |  | unknown |
| 12 | CG17127 | CG17127 |  | CG17127 | FBgn0032299 | Unrelated | Cuticle | unknown |
| 13 | Syt7 | Syt7 | Synaptotagmin 7 | CG2381 | FBgn0039900 | Membrane |  | calcium-dependent phospholipid binding |
| 14 | mld | Mld | Molting defective | CG34100 | FBgn0263490 | Other |  | zinc ion binding; nucleic acid binding |
| 15 | htl | FGFR | Heartless | CG7223 | FBgn0010389 | G-protein | Membrane | protein tyrosine kinase activity;  fibroblast growth factor-activated receptor activity |
| 16 | CG15701 | CG15701 | CG15701 | CG15701 | FBgn0034095 | Unknown |  | unknown |
| 17 | AGO2 | AGO2 | Argonaute 2 | CG7439 | FBgn0087035 | RNA |  | protein binding; endoribonuclease activity; siRNA binding |
| 18 | tyn | Trynity | Trynity | CG17131 | FBgn0029128 | Membrane | Cytoskeleton | unknown |
| 19 | snf | SNF | Sans fille | CG4528 | FBgn0003449 | RNA |  | U2 snRNA binding; protein binding; snRNA stem-loop binding;  U1 snRNA binding |
| 20 | mfrn | Mitoferrin | Mitoferrin | CG4963 | FBgn0039561 | Membrane |  | iron ion transmembrane transporter activity |
| 21 | CG17271 | CG17271 | CG17271 | CG17271 | FBgn0038829 | Unknown |  | calcium ion binding |
| 22 | Tequila | Tequila | Tequila | CG4821 | FBgn0023479 | Membrane |  | serine-type endopeptidase activity |
| 23 | Rab3-GEF | Rab3-GEF | Rab3 GDP-GTP exchange factor | CG5627 | FBgn0030613 | G-protein | Membrane | Rab guanyl-nucleotide exchange factor activity |
| 24 | Cpr65Av | Cpr65Av | Cuticular protein 65Av | CG32405 | FBgn0052405 | Unrelated | Cuticle | structural constituent of chitin-based cuticle |
| 25 | SF1 | SF1 | Splicing factor 1 | CG5836 | FBgn0025571 | RNA |  | RNA binding; zinc ion binding |
| 26 | Saf-B | SAF-B | Scaffold attachment factor B | CG6995 | FBgn0039229 | RNA |  | mRNA binding |
| 27 | NAT1 | NAT1 | NAT1 | CG3845 | FBgn0010488 | RNA |  | translation initiation factor activity |
| 28 | Con | Connectin | Connectin | CG7503 | FBgn0005775 | Membrane |  | unknown |
| 29 | Lcp65Ad | Lcp65Ad | Larval cuticular protein 65Ad | CG6955 | FBgn0020641 | Unrelated | Cuticle | structural constituent of chitin-based cuticle |
| 30 | His3:CG31613 | Histone 3 | His3:CG31613 | CG31613 | FBgn0051613 | Other |  | DNA binding |
| 31 | Cdep | CDEP | Chondrocyte-derived ezrin-like domain containing protein ortholog | CG44193 | FBgn0265082 | G-protein | Cytoskeleton | actin binding; Rho guanyl-nucleotide exchange factor activity |
| 32 | CG10625 | CG10625 |  | CG10625 | FBgn0035612 | Unrelated | Cuticle | structural constituent of cuticle |
| 33 | Zasp52 | Zasp | Z band alternatively spliced  PDZ-motif protein 52 | CG30084 | FBgn0083919 | Other |  | protein binding |
| 34 | CG7903 | CG7903 | CG7903 | CG7903 | FBgn0039730 | RNA |  | mRNA binding |
| 35 | CG31619 | CG31619 | CG31619 | CG31619 | FBgn0051619 | Membrane |  | metalloendopeptidase activity |
| 36 | Nup43 | Nup43 | Nucleoporin 43kD | CG7671 | FBgn0038609 | Membrane |  | unknown |
| 37 | Moca-cyp | Moca-cyp | Moca-cyp | CG1866 | FBgn0039581 | RNA |  | peptidyl-prolyl cis-trans isomerase activity |
