# Supplementary material for: Drep-2 is a novel synaptic protein important for learning and memory
Source: eLife. 2014 Nov 13;3:e03895. doi: 10.7554/eLife.03895 (PMC4229683; doi:10.7554/eLife.03895)
Supplement: Supplementary file 2. — Mass spectrometry: all proteins enriched at an FDR of 10%. The table shows two ratios for all proteins: first, Drep-2GFP animals, GFP beads vs. plain beads and, second, GFP beads, Drep-2GFP vs. wild-type (wt) animals (compare Figure 7A). “+” symbols indicate whether a protein was significantly enriched at a given FDR. Proteins are sorted according to the first ratio. Proteins were labeled in cyan if significantly enriched for both ratios at an FDR of 1%; these proteins constitute the core interactors. Protein names were labeled in red if the protein was positively enriched for the first ratio but negatively enriched for the second one, and thus probably constitutes a false positive. Protein names were labeled in shades of green if the protein was included as an additional interactor in the network of interacting proteins (Figure 7D): dark green = FDR 1%, medium green = FDR 5%, light green = FDR 10%. DOI: http://dx.doi.org/10.7554/eLife.03895.016 [file elife03895s002.docx]

| **Rank** | **Flybase name** | **Name in network** | **CG number** | **FDR 1%,**  **both ratios** | **Ratio**  **GFP vs. plain** | **FDR 1% (GFP vs. plain)** | **FDR 5% (GFP vs. plain)** | **FDR 10% (GFP vs. plain)** | **Ratio**  **Drep-2^GFP^**  **vs. wt** | **FDR 1% (Drep-2^GFP^ vs. wt)** | **FDR 10% (Drep-2^GFP^ vs. wt)** |
| --- | --- | --- | --- | --- | --- | --- | --- | --- | --- | --- | --- |
| 1 | crb | Crumbs | CG6383 | + | 12.3250 | + | + | + | 10.7500 | + | + |
| 2 | cos | Cos2 | CG1708 | + | 10.3748 | + | + | + | 10.4311 | + | + |
| 3 | sif | SIF | CG34418 | + | 8.4918 | + | + | + | 2.4731 | + | + |
| 4 | Capr | Caprin | CG18811 | + | 7.6650 | + | + | + | 9.0782 | + | + |
| 5 | mbo | Nup88 | CG6819 | + | 7.4674 | + | + | + | 7.7013 | + | + |
| 6 | GFP |  | GFP | + | 7.2607 | + | + | + | 15.9107 | + | + |
| 7 | Drep-2 | Drep-2 | CG1975 | + | 7.1429 | + | + | + | 6.5453 | + | + |
| 8 | CG14095 | CG14095 | CG14095 | + | 7.0700 | + | + | + | 3.6235 | + | + |
| 9 | RnpS1 | RnpS1 | CG16788 | + | 7.0348 | + | + | + | 7.6030 | + | + |
| 10 | CG30122 | hnRNP U | CG30122 | + | 6.9177 | + | + | + | 2.8685 | + | + |
| 11 | Drep-3 | Drep-3 | CG8364 | + | 6.3578 | + | + | + | 6.2338 | + | + |
| 12 | CG17127 | CG17127 | CG17127 | + | 6.1355 | + | + | + | 3.4957 | + | + |
| 13 | Syt7 | Syt7 | CG2381 | + | 5.8968 | + | + | + | 5.8019 | + | + |
| 14 | mld | Mld | CG34100 | + | 5.8475 | + | + | + | 5.3062 | + | + |
| 15 | htl | FGFR | CG7223 | + | 5.8126 | + | + | + | 6.8626 | + | + |
| 16 | CG15701 | CG15701 | CG15701 | + | 5.2069 | + | + | + | 4.1492 | + | + |
| 17 | AGO2 | AGO2 | CG7439 | + | 5.1545 | + | + | + | 4.2895 | + | + |
| 18 | tyn | Trynity | CG17131 | + | 5.1305 | + | + | + | 5.0728 | + | + |
| 19 | snf | SNF | CG4528 | + | 5.0372 | + | + | + | 4.7603 | + | + |
| 20 | mfrn | Mitoferrin | CG4963 | + | 4.9878 | + | + | + | 4.5722 | + | + |
| 21 | CG17271 | CG17271 | CG17271 | + | 4.9798 | + | + | + | 4.2631 | + | + |
| 22 | Tequila | Tequila | CG4821 | + | 4.6810 | + | + | + | 5.3703 | + | + |
| 23 | Rab3-GEF | Rab3-GEF | CG5627 | + | 4.6239 | + | + | + | 3.9924 | + | + |
| 24 | Cpr65Av | Cpr65Av | CG32405 | + | 4.5688 | + | + | + | 3.9534 | + | + |
| 25 | SF1 | SF1 | CG5836 | + | 4.5524 | + | + | + | 3.6150 | + | + |
| 26 | Saf-B | SAF-B | CG6995 | + | 4.4753 | + | + | + | 4.0609 | + | + |
| 27 | NAT1 | NAT1 | CG3845 | + | 4.3966 | + | + | + | 5.3712 | + | + |
| 28 | Con | Connectin | CG7503 | + | 4.3827 | + | + | + | 3.8714 | + | + |
| 29 | Lcp65Ad | Lcp65Ad | CG6955 | + | 4.1727 | + | + | + | 4.3942 | + | + |
| 30 | His3:CG31613 | Histone 3 | CG31613 | + | 4.0437 | + | + | + | 4.0750 | + | + |
| 31 | Cdep | CDEP | CG44193 | + | 3.9467 | + | + | + | 4.1342 | + | + |
| 32 | CG10625 | CG10625 | CG10625 | + | 3.9061 | + | + | + | 4.4861 | + | + |
| 33 | Zasp52 | Zasp | CG30084 | + | 3.8798 | + | + | + | 4.9279 | + | + |
| 34 | CG7903 | CG7903 | CG7903 | + | 3.5561 | + | + | + | 3.0366 | + | + |
| 35 | CG31619 | CG31619 | CG31619 | + | 3.4817 | + | + | + | 2.6705 | + | + |
| 36 | Nup43 | Nup43 | CG7671 | + | 3.4291 | + | + | + | 4.0497 | + | + |
| 37 | Moca-cyp | Moca-cyp | CG1866 | + | 3.3678 | + | + | + | 4.3624 | + | + |
| 38 | CG30101 |  | CG30101 |  | 10.0942 | + | + | + | -2.3857 |  | - |
| 39 | Prm |  | CG5939 |  | 9.4515 | + | + | + | -1.5074 |  | - |
| 40 | TwdlT |  | CG5812 |  | 9.3754 | + | + | + | -0.5081 |  |  |
| 41 | CG4962 |  | CG4962 |  | 8.7779 | + | + | + | -1.3405 |  | - |
| 42 | CG16886 |  | CG16886 |  | 8.7611 | + | + | + | -2.8201 | - | - |
| 43 | Cpr62Bb |  | CG13935 |  | 8.4752 | + | + | + | -1.2808 |  |  |
| 44 | CG8192 |  | CG8192 |  | 8.3554 | + | + | + | -1.1825 |  |  |
| 45 | CG2150 |  | CG2150 |  | 8.1571 | + | + | + | -0.6762 |  |  |
| 46 | resilin |  | CG15920 |  | 8.1539 | + | + | + | 0.1276 |  |  |
| 47 | Cpr73D |  | CG9665 |  | 8.0930 | + | + | + | -1.2242 |  |  |
| 48 | CG17298 |  | CG17298 |  | 7.7924 | + | + | + | -1.0912 |  |  |
| 49 | CG13071 |  | CG13071 |  | 7.7548 | + | + | + | -3.0470 | - | - |
| 50 | Cpr78Cc |  | CG7658 |  | 7.7489 | + | + | + | -3.0832 | - | - |
| 51 | Cpr57A |  | CG18066 |  | 7.6800 | + | + | + | -2.0463 |  | - |
| 52 | Cpr92F |  | CG5494 |  | 7.6610 | + | + | + | -1.4905 |  | - |
| 53 | Cpr49Ag |  | CG8511 |  | 7.5948 | + | + | + | -0.3914 |  |  |
| 54 | Ccp84Ag |  | CG2342 |  | 7.5481 | + | + | + | -1.0665 |  |  |
| 55 | Zasp52 |  | CG30084 |  | 7.3669 | + | + | + | -1.6432 |  | - |
| 56 | CG13022 |  | CG13022 |  | 7.3219 | + | + | + | -0.3281 |  |  |
| 57 | CG14712 |  | CG14712 |  | 7.2567 | + | + | + | 1.1301 |  |  |
| 58 | CG13044 |  | CG13044 |  | 7.2556 | + | + | + | 3.1750 |  | + |
| 59 | Act88F |  | CG5178 |  | 7.2326 | + | + | + | -3.5509 | - | - |
| 60 | Ccp84Ae |  | CG1330 |  | 7.1735 | + | + | + | -0.0316 |  |  |
| 61 | CG13043 |  | CG13043 |  | 7.1353 | + | + | + | 3.2718 |  |  |
| 62 | CG6055 |  | CG6055 |  | 7.1269 | + | + | + | -1.9629 |  | - |
| 63 | Cpr50Cb |  | CG6305 |  | 7.0354 | + | + | + | 1.5286 |  |  |
| 64 | CG43897 |  | CG33205 |  | 6.8588 | + | + | + | -1.1276 |  |  |
| 65 | Pxd |  | CG3477 |  | 6.8586 | + | + | + | 1.6755 |  | + |
| 66 | l(3)mbn |  | CG12755 |  | 6.7308 | + | + | + | -0.2835 |  |  |
| 67 | Cg25C |  | CG4145 |  | 6.5213 | + | + | + | -1.1333 |  |  |
| 68 | zye |  | CG5847 |  | 6.4370 | + | + | + | 2.2532 |  |  |
| 69 | CG13060 |  | CG13060 |  | 6.3821 | + | + | + | 3.5024 |  |  |
| 70 | Cpr62Bc |  | CG1919 |  | 6.3747 | + | + | + | -0.9298 |  |  |
| 71 | Cpr76Bd |  | CG9299 |  | 6.3325 | + | + | + | -1.7203 |  | - |
| 72 | Pcp |  | CG3440 |  | 6.2415 | + | + | + | -1.3420 |  | - |
| 73 | CG14752 |  | CG14752 |  | 6.2215 | + | + | + | -2.8193 | - | - |
| 74 | CG7409 |  | CG7409 |  | 6.1810 | + | + | + | -0.3385 |  |  |
| 75 | Cpr72Ec |  | CG4784 |  | 6.1634 | + | + | + | -0.8205 |  |  |
| 76 | Cpr64Ab |  | CG15007 |  | 6.1539 | + | + | + | -1.3013 |  | - |
| 77 | Cpr30F |  | CG31876 |  | 6.1457 | + | + | + | -2.5347 |  | - |
| 78 | Hrb87F | hnRNP 36 | CG12749 |  | 6.0591 | + | + | + | 2.4026 |  |  |
| 79 | qkr58E-1 | Qkr58E-1 | CG3613 |  | 6.0506 | + | + | + | 3.3310 |  | + |
| 80 | Mhc |  | CG17927 |  | 5.9441 | + | + | + | 0.6369 |  |  |
| 81 | CG9572 |  | CG9572 |  | 5.9423 | + | + | + | -1.2119 |  |  |
| 82 | Rsf1 |  | CG5655 |  | 5.9410 | + | + | + | 3.3110 |  | + |
| 83 | Cpr67B |  | CG3672 |  | 5.9142 | + | + | + | -0.8259 |  |  |
| 84 | CG16885 |  | CG16885 |  | 5.8960 | + | + | + | -1.4247 |  | - |
| 85 | Mhc |  | CG17927 |  | 5.8925 |  | + | + | -1.8581 |  | - |
| 86 | yellow-e |  | CG9792 |  | 5.8610 | + | + | + | -1.5966 |  | - |
| 87 | by | Tensin | CG9379 |  | 5.8544 | + | + | + | 0.0702 |  |  |
| 88 | Cry |  | CG16963 |  | 5.8273 | + | + | + | -0.5529 |  |  |
| 89 | CG16884 |  | CG16884 |  | 5.7707 | + | + | + | -0.9479 |  |  |
| 90 | CG8547 |  | CG8547 |  | 5.7198 | + | + | + | 0.8531 |  |  |
| 91 | Cpr47Ee |  | CG13222 |  | 5.7063 | + | + | + | 0.4954 |  |  |
| 92 | Cpr47Ef |  | CG13214 |  | 5.7024 | + | + | + | 1.4094 |  |  |
| 93 | CG8929 |  | CG8929 |  | 5.6624 | + | + | + | -2.3339 | - | - |
| 94 | Cpr64Ad |  | CG1259 |  | 5.6334 | + | + | + | -0.1021 |  |  |
| 95 | Edg78E |  | CG7673 |  | 5.6290 | + | + | + | 1.0730 |  |  |
| 96 | Ccp84Ab |  | CG1252 |  | 5.5782 | + | + | + | 0.6860 |  |  |
| 97 | LanB1 |  | CG7123 |  | 5.5328 | + | + | + | -0.5609 |  |  |
| 98 | Unc-89 |  | CG33519 |  | 5.5116 | + | + | + | -1.2035 |  |  |
| 99 | CAP |  | CG18408 |  | 5.4469 | + | + | + | -0.7460 |  |  |
| 100 | CG15515 |  | CG15515 |  | 5.4312 | + | + | + | 1.2320 |  |  |
| 101 | sls |  | CG1915 |  | 5.3615 | + | + | + | -1.2641 |  |  |
| 102 | CG13065 |  | CG13065 |  | 5.3333 | + | + | + | -0.4716 |  |  |
| 103 | CG13026 |  | CG13026 |  | 5.3244 | + | + | + | 0.7437 |  |  |
| 104 | CG42367 |  | CG42367 |  | 5.3044 | + | + | + | -0.7208 |  |  |
| 105 | CG32694 |  | CG32694 |  | 5.2891 | + | + | + | -1.1535 |  |  |
| 106 | CG34461 |  | CG34461 |  | 5.2650 | + | + | + | -0.7756 |  |  |
| 107 | Zasp66 |  | CG6416 |  | 5.2275 |  | + | + | -2.5140 |  | - |
| 108 | vkg |  | CG16858 |  | 5.2015 | + | + | + | -1.3143 |  | - |
| 109 | Prm |  | CG5939 |  | 5.1924 | + | + | + | -0.4371 |  |  |
| 110 | Cpr49Aa |  | CG30045 |  | 5.1714 | + | + | + | -0.7920 |  |  |
| 111 | CG7587 |  | CG7587 |  | 5.1565 | + | + | + | -2.0063 |  | - |
| 112 | scaf |  | CG11066 |  | 5.1389 | + | + | + | -1.2457 |  |  |
| 113 | Hrb98DE | hnRNP 38 | CG9983 |  | 5.1212 | + | + | + | 0.8364 |  |  |
| 114 | Rm62 |  | CG10279 |  | 5.1175 | + | + | + | 0.7100 |  |  |
| 115 | CG3689 | CG3689 | CG3689 |  | 5.0931 | + | + | + | 0.6320 |  |  |
| 116 | Cpr49Ac |  | CG8502 |  | 4.9889 | + | + | + | 1.2221 |  |  |
| 117 | CG10359 |  | CG10359 |  | 4.9428 | + | + | + | -1.5204 |  | - |
| 118 | Glt |  | CG9280 |  | 4.8943 | + | + | + | 0.3813 |  |  |
| 119 | Cpr49Ae |  | CG8505 |  | 4.8814 | + | + | + | -0.0818 |  |  |
| 120 | Lcp65Ac |  | CG6956 |  | 4.8768 | + | + | + | -1.0207 |  |  |
| 121 | CG7214 |  | CG7214 |  | 4.8747 | + | + | + | 0.4479 |  |  |
| 122 | Nup98-96 | Nup98 | CG10198 |  | 4.8721 |  | + | + | 4.4499 | + | + |
| 123 | CG32335 |  | CG32335 |  | 4.8585 | + | + | + | 4.2772 |  | + |
| 124 | fau |  | CG6544 |  | 4.8375 | + | + | + | -0.6076 |  |  |
| 125 | CG34247 |  | CG34247 |  | 4.8365 | + | + | + | 0.1623 |  |  |
| 126 | Cpr64Ac |  | CG15008 |  | 4.8245 | + | + | + | -2.2486 |  | - |
| 127 | CG13049 |  | CG13049 |  | 4.8175 | + | + | + | 0.9359 |  |  |
| 128 | Acp65Aa |  | CG10297 |  | 4.7514 | + | + | + | 1.2199 |  |  |
| 129 | mud |  | CG12047 |  | 4.7358 |  | + | + | 5.3256 |  | + |
| 130 | Mlc2 |  | CG2184 |  | 4.6391 | + | + | + | -0.6817 |  |  |
| 131 | CG42323 |  | CG42323 |  | 4.5923 | + | + | + | -1.8279 |  | - |
| 132 | retinin |  | CG13057 |  | 4.5757 | + | + | + | -1.5813 |  | - |
| 133 | CG13422 |  | CG13422 |  | 4.5545 | + | + | + | 1.1686 |  |  |
| 134 | CG5172 |  | CG5172 |  | 4.5462 | + | + | + | 0.1371 |  |  |
| 135 | Tm1 |  | CG4898 |  | 4.5352 | + | + | + | -1.7756 |  | - |
| 136 | CG13841 |  | CG13841 |  | 4.5147 | + | + | + | -2.5537 | - | - |
| 137 | CG9492 |  | CG9492 |  | 4.4935 | + | + | + | -0.8267 |  |  |
| 138 | CG13056 |  | CG13056 |  | 4.4787 | + | + | + | -2.7394 | - | - |
| 139 | CG5787 |  | CG5787 |  | 4.4787 |  | + | + | 4.4463 | + | + |
| 140 | Mf |  | CG6803 |  | 4.4589 | + | + | + | -1.0718 |  |  |
| 141 | Cpr64Aa |  | CG15006 |  | 4.4445 | + | + | + | -1.2303 |  | - |
| 142 | Cpr65Au |  | CG18778 |  | 4.4248 | + | + | + | -1.6193 |  | - |
| 143 | CG34205 |  | CG34205 |  | 4.4174 | + | + | + | -2.9039 | - | - |
| 144 | Cpr66D |  | CG32029 |  | 4.3484 | + | + | + | -0.7816 |  |  |
| 145 | Mhc |  | CG17927 |  | 4.3453 | + | + | + | -1.0859 |  |  |
| 146 | Skeletor |  | CG14682 |  | 4.3440 |  | + | + | 4.8253 | + | + |
| 147 | Cpr47Ea |  | CG9079 |  | 4.3388 | + | + | + | -0.3678 |  |  |
| 148 | CG17549 |  | CG17549 |  | 4.3290 |  | + | + | 1.5277 |  |  |
| 149 | CG5001 | CG5001 | CG5001 |  | 4.3121 |  | + | + | 2.6387 | + | + |
| 150 | Lcp65Ag1 |  | CG10530 |  | 4.3071 | + | + | + | -2.3130 |  | - |
| 151 | CG15772 |  | CG15772 |  | 4.2786 | + | + | + | 1.5514 |  |  |
| 152 | Tm2 |  | CG4843 |  | 4.2629 | + | + | + | -1.0846 |  |  |
| 153 | x16 | X16 | CG10203 |  | 4.2287 | + | + | + | 0.4443 |  |  |
| 154 | Mhc |  | CG17927 |  | 4.2212 | + | + | + | -1.3832 |  | - |
| 155 | hig | Hig | CG2040 |  | 4.1957 | + | + | + | 2.7272 |  |  |
| 156 | slik |  | CG4527 |  | 4.1832 | + | + | + | -2.7019 | - | - |
| 157 | mRpL1 |  | CG7494 |  | 4.1807 | + | + | + | 3.4671 |  | + |
| 158 | Tm2 |  | CG4843 |  | 4.1545 | + | + | + | -1.5590 |  | - |
| 159 | LanB2 |  | CG3322 |  | 4.1441 | + | + | + | 0.1111 |  |  |
| 160 | His2Av |  | CG5499 |  | 4.1379 | + | + | + | -0.6289 |  |  |
| 161 | obst-E |  | CG11142 |  | 4.1135 |  | + | + | 0.5181 |  |  |
| 162 | wupA |  | CG7178 |  | 4.0794 | + | + | + | -2.0012 |  | - |
| 163 | Acp1 |  | CG7216 |  | 4.0789 | + | + | + | 0.2729 |  |  |
| 164 | Cpr97Eb |  | CG15884 |  | 4.0564 | + | + | + | -0.5689 |  |  |
| 165 | Bin1 | SAP18 | CG6046 |  | 4.0549 | + | + | + | 2.6434 |  | + |
| 166 | Cpr100A |  | CG12045 |  | 4.0429 | + | + | + | -0.1747 |  |  |
| 167 | qsm |  | CG13432 |  | 4.0288 |  | + | + | 3.3595 | + | + |
| 168 | CG2157 |  | CG2157 |  | 3.9684 | + | + | + | -2.4218 | - | - |
| 169 | His4:CG31611 |  | CG31611 |  | 3.9574 | + | + | + | -0.0371 |  |  |
| 170 | cpb |  | CG17158 |  | 3.9406 |  | + | + | 0.6255 |  |  |
| 171 | mRpL41 |  | CG12954 |  | 3.9401 | + | + | + | -0.2471 |  |  |
| 172 | CG13841 |  | CG13841 |  | 3.9319 |  | + | + | -0.3743 |  |  |
| 173 | CG7185 | CG7185 | CG7185 |  | 3.8856 | + | + | + | 2.3518 |  | + |
| 174 | Peritrophin-A |  | CG17058 |  | 3.8705 | + | + | + | -2.0528 |  | - |
| 175 | His2B:CG17949 |  | CG17949 |  | 3.8662 | + | + | + | 0.5336 |  |  |
| 176 | PI4KIIIalpha |  | CG10260 |  | 3.8548 | + | + | + | 3.1204 |  | + |
| 177 | cindr |  | CG31012 |  | 3.8353 |  | + | + | 5.6099 | + | + |
| 178 | His3.3A |  | CG5825 |  | 3.8348 | + | + | + | 0.1541 |  |  |
| 179 | sls |  | CG1915 |  | 3.8250 |  | + | + | -0.8958 |  |  |
| 180 | D1 |  | CG9745 |  | 3.8178 | + | + | + | 5.3722 |  | + |
| 181 | up |  | CG7107 |  | 3.8098 | + | + | + | -1.1136 |  |  |
| 182 | Act87E |  | CG18290 |  | 3.7985 | + | + | + | -2.8852 | - | - |
| 183 | CG4461 |  | CG4461 |  | 3.7819 |  |  | + | -0.2479 |  |  |
| 184 | CkIIbeta | CK II | CG15224 |  | 3.7806 | + | + | + | -1.1669 |  |  |
| 185 | Mf |  | CG6803 |  | 3.7658 | + | + | + | -1.4350 |  | - |
| 186 | Tm1 |  | CG4898 |  | 3.6996 | + | + | + | -0.2338 |  |  |
| 187 | Cpr49Ab |  | CG30042 |  | 3.6826 | + | + | + | -1.4567 |  |  |
| 188 | nonA |  | CG4211 |  | 3.6491 | + | + | + | 1.2324 |  |  |
| 189 | Zasp66 |  | CG6416 |  | 3.6484 | + | + | + | -0.8886 |  |  |
| 190 | qkr58E-2 | Qkr58E-2 | CG5821 |  | 3.5943 |  |  | + | 1.8320 |  |  |
| 191 | Msr-110 |  | CG10596 |  | 3.5897 |  | + | + | 2.5263 |  | + |
| 192 | Rbp |  | CG31302 |  | 3.5416 |  | + | + | 0.4483 |  |  |
| 193 | Ndg |  | CG12908 |  | 3.4918 | + | + | + | -0.3820 |  |  |
| 194 | His2A:CG31618 |  | CG31618 |  | 3.4799 | + | + | + | 0.7809 |  |  |
| 195 | CASK |  | CG6703 |  | 3.4529 |  | + | + | 2.5232 |  | + |
| 196 | Strn-Mlck |  | CG18255 |  | 3.4478 |  |  | + | 0.2201 |  |  |
| 197 | koi |  | CG3287 |  | 3.4414 | + | + | + | 0.0753 |  |  |
| 198 | Mlc1 |  | CG5596 |  | 3.4368 | + | + | + | -0.8493 |  |  |
| 199 | wupA |  | CG7178 |  | 3.4259 | + | + | + | -1.6568 |  | - |
| 200 | Srp54 | Srp54 | CG4602 |  | 3.4124 |  | + | + | 2.4501 |  | + |
| 201 | Mhc |  | CG17927 |  | 3.4060 |  | + | + | -1.5145 |  |  |
| 202 | glo | Glorund | CG6946 |  | 3.3968 | + | + | + | 1.3708 |  | + |
| 203 | Cpr51A |  | CG10112 |  | 3.3669 | + | + | + | 0.4032 |  |  |
| 204 | bel |  | CG9748 |  | 3.3474 | + | + | + | 1.3631 |  |  |
| 205 | Hsp70Ab |  | CG18743 |  | 3.2973 |  |  | + | 2.9422 |  |  |
| 206 | CG43078 |  | CG32352 |  | 3.2945 |  | + | + | 2.6979 |  | + |
| 207 | CG2010 |  | CG2010 |  | 3.2766 |  | + | + | 3.5158 | + | + |
| 208 | Akap200 |  | CG13388 |  | 3.2660 |  | + | + | 0.7101 |  |  |
| 209 | tral |  | CG10686 |  | 3.2515 | + | + | + | 4.4847 |  | + |
| 210 | LanA |  | CG10236 |  | 3.2194 | + | + | + | -0.1993 |  |  |
| 211 | wupA |  | CG7178 |  | 3.2174 | + | + | + | -2.6158 | - | - |
| 212 | up |  | CG7107 |  | 3.2083 |  | + | + | -2.4380 |  | - |
| 213 | Su(var)205 |  | CG8409 |  | 3.2041 | + | + | + | -0.4056 |  |  |
| 214 | Cpr47Eg |  | CG9070 |  | 3.1431 |  | + | + | -2.2710 |  |  |
| 215 | Cpr65Ax2 |  | CG18777 |  | 3.1412 | + | + | + | 3.4979 |  | + |
| 216 | hts |  | CG9325 |  | 3.1186 |  | + | + | -0.7165 |  |  |
| 217 | snRNPU1-70K | snRNP U1 | CG8749 |  | 3.1161 |  | + | + | 2.9771 |  | + |
| 218 | CG32082 |  | CG32082 |  | 3.1041 |  | + | + | -0.0719 |  |  |
| 219 | U2af38 | U2-AF 38 | CG3582 |  | 3.0805 |  | + | + | 3.7231 | + | + |
| 220 | CG42450 |  | CG42450 |  | 3.0447 |  | + | + | 3.6504 | + | + |
| 221 | fau |  | CG6544 |  | 3.0268 | + | + | + | 0.3242 |  |  |
| 222 | Ama |  | CG2198 |  | 3.0212 |  |  | + | 1.4162 |  |  |
| 223 | TpnC47D |  | CG9073 |  | 3.0199 |  | + | + | 1.1546 |  |  |
| 224 | CG5873 |  | CG5873 |  | 3.0077 |  | + | + | -3.3521 | - | - |
| 225 | CG10777 |  | CG10777 |  | 2.9935 |  |  | + | 6.4281 | + | + |
| 226 | Mf |  | CG6803 |  | 2.9767 |  | + | + | 2.5025 |  |  |
| 227 | CG34172 |  | CG34172 |  | 2.9666 | + | + | + | 3.0198 |  |  |
| 228 | Rya-r44F |  | CG10844 |  | 2.9370 |  |  | + | -0.1908 |  |  |
| 229 | Fmr1 | FMRP | CG6203 |  | 2.9184 |  | + | + | 3.3729 |  | + |
| 230 | Cpr49Ah |  | CG8515 |  | 2.8829 | + | + | + | -1.2436 |  |  |
| 231 | Ncc69 |  | CG4357 |  | 2.8772 |  | + | + | 2.6637 |  | + |
| 232 | CG31345 |  | CG31345 |  | 2.8767 |  | + | + | 2.9714 |  |  |
| 233 | Mad1 |  | CG2072 |  | 2.8731 |  | + | + | 2.2341 |  | + |
| 234 | CG42863 |  | CG11814 |  | 2.8281 |  |  | + | -5.5518 |  | - |
| 235 | rump | hnRNP M | CG9373 |  | 2.8188 |  | + | + | -0.0756 |  |  |
| 236 | CG13840 |  | CG13840 |  | 2.8094 |  |  | + | -3.0508 |  |  |
| 237 | Act57B |  | CG10067 |  | 2.7824 | + | + | + | -0.8573 |  |  |
| 238 | Cpr66Cb |  | CG7076 |  | 2.7771 |  |  | + | -1.1917 |  |  |
| 239 | shg |  | CG3722 |  | 2.7609 |  | + | + | 4.2574 | + | + |
| 240 | CG30159 |  | CG30159 |  | 2.7433 |  |  | + | -0.4701 |  |  |
| 241 | CG8632 |  | CG8632 |  | 2.7171 |  | + | + | 2.0037 |  |  |
| 242 | fau |  | CG6544 |  | 2.7023 | + | + | + | 1.1714 |  |  |
| 243 | His1:CG31617 |  | CG31617 |  | 2.6785 | + | + | + | -0.1903 |  |  |
| 244 | Zasp52 | ZasP52 | CG30084 |  | 2.6777 | + | + | + | -1.1991 |  |  |
| 245 | Nup75 | Nup75 | CG5733 |  | 2.6447 |  | + | + | 1.8909 |  |  |
| 246 | Pur-alpha |  | CG1507 |  | 2.6426 |  | + | + | 3.0714 |  | + |
| 247 | sxc |  | CG10392 |  | 2.6353 |  |  | + | 2.5760 |  |  |
| 248 | Mhc |  | CG17927 |  | 2.6300 |  |  | + | -7.5077 | - | - |
| 249 | Gmap |  | CG33206 |  | 2.6296 |  | + | + | 5.3133 | + | + |
| 250 | laccase2 |  | CG30437 |  | 2.6279 | + | + | + | -0.8951 |  |  |
| 251 | CG13096 |  | CG13096 |  | 2.6184 |  | + | + | 4.0963 | + | + |
| 252 | CG2310 |  | CG2310 |  | 2.6156 |  | + | + | 1.6031 |  |  |
| 253 | CG12333 |  | CG12333 |  | 2.6091 |  | + | + | 1.8263 |  |  |
| 254 | vir-1 |  | CG31764 |  | 2.6066 |  | + | + | 3.8061 |  | + |
| 255 | CG8736 |  | CG8736 |  | 2.6045 |  | + | + | -1.9013 |  | - |
| 256 | CG8108 |  | CG8108 |  | 2.5846 |  | + | + | 1.9799 |  | + |
| 257 | trol |  | CG33950 |  | 2.5820 | + | + | + | -1.0637 |  |  |
| 258 | l(2)k14710 |  | CG8325 |  | 2.5722 |  | + | + | 3.6975 | + | + |
| 259 | Actn |  | CG4376 |  | 2.5456 | + | + | + | -1.3008 |  | - |
| 260 | Nop56 |  | CG13849 |  | 2.5451 |  |  | + | 3.9108 | + | + |
| 261 | CG7781 |  | CG7781 |  | 2.5405 |  | + | + | -1.3797 |  | - |
| 262 | wupA |  | CG7178 |  | 2.5349 |  | + | + | -0.8004 |  |  |
| 263 | TpnC25D |  | CG6514 |  | 2.5212 | + | + | + | -1.1289 |  |  |
| 264 | Prp8 | PRP8 | CG8877 |  | 2.4797 |  |  | + | 1.2826 |  |  |
| 265 | GNBP3 |  | CG5008 |  | 2.4745 | + | + | + | 3.9542 |  | + |
| 266 | Msp-300 |  | CG33715 |  | 2.4615 | + | + | + | 0.2666 |  |  |
| 267 | CG31321 |  | CG31321 |  | 2.4529 |  | + | + | -2.3521 |  | - |
| 268 | bt |  | CG32019 |  | 2.4254 | + | + | + | -1.2020 |  |  |
| 269 | Gycbeta100B |  | CG1470 |  | 2.4078 |  | + | + | 1.5753 |  |  |
| 270 | obst-E |  | CG11142 |  | 2.3847 |  |  | + | -1.4925 |  |  |
| 271 | e(r) |  | CG1871 |  | 2.3792 |  |  | + | 2.3686 |  |  |
| 272 | TpnC73F |  | CG7930 |  | 2.3755 |  | + | + | -0.7026 |  |  |
| 273 | CG11147 |  | CG11147 |  | 2.3656 |  | + | + | 0.9048 |  |  |
| 274 | brp |  | CG42344 |  | 2.3604 |  | + | + | -0.3332 |  |  |
| 275 | rhea |  | CG6831 |  | 2.3493 |  | + | + | -0.3896 |  |  |
| 276 | U2A | U2A | CG1406 |  | 2.3481 |  | + | + | 1.4691 |  |  |
| 277 | RSG7 |  | CG9108 |  | 2.3438 |  | + | + | 1.7450 |  | + |
| 278 | Hsp67Bc |  | CG4190 |  | 2.3427 |  | + | + | 5.7351 |  | + |
| 279 | CG6347 |  | CG6347 |  | 2.3425 |  | + | + | 2.4742 |  | + |
| 280 | SmF | SmF | CG16792 |  | 2.3350 |  | + | + | 5.7029 | + | + |
| 281 | mRpS16 |  | CG8338 |  | 2.3262 |  |  | + | 2.6076 |  | + |
| 282 | Actn |  | CG4376 |  | 2.3252 |  | + | + | -0.9724 |  |  |
| 283 | tmod |  | CG1539 |  | 2.2929 |  | + | + | -0.7050 |  |  |
| 284 | nocte |  | CG17255 |  | 2.2922 |  | + | + | 3.1926 | + | + |
| 285 | LBR |  | CG17952 |  | 2.2817 |  | + | + | 3.0120 |  | + |
| 286 | CG6406 |  | CG6406 |  | 2.2749 |  |  | + | 2.6018 |  | + |
| 287 | CG13900 | CG13900 | CG13900 |  | 2.2658 |  | + | + | 2.0123 |  | + |
| 288 | cm |  | CG3035 |  | 2.2579 |  | + | + | 2.6385 | + | + |
| 289 | Tsp |  | CG11326 |  | 2.2321 |  | + | + | 2.6209 |  |  |
| 290 | cype |  | CG14028 |  | 2.2261 |  | + | + | 0.0802 |  |  |
| 291 | AP-2sigma |  | CG6056 |  | 2.1997 |  | + | + | 0.5298 |  |  |
| 292 | CG15117 |  | CG15117 |  | 2.1881 |  | + | + | 0.5271 |  |  |
| 293 | CG30069 |  | CG30069 |  | 2.1833 |  | + | + | 8.0665 | + | + |
| 294 | rin |  | CG9412 |  | 2.1782 |  | + | + | 2.5182 |  | + |
| 295 | LamC |  | CG10119 |  | 2.1696 |  | + | + | -0.1383 |  |  |
| 296 | 7B2 |  | CG1168 |  | 2.1654 |  | + | + | 2.0360 |  | + |
| 297 | Dic1 |  | CG8790 |  | 2.1614 |  | + | + | 1.3199 |  |  |
| 298 | Fit2 |  | CG7729 |  | 2.1560 |  |  | + | 2.4772 |  | + |
| 299 | att-ORFA |  | CG4241 |  | 2.1553 |  |  | + | 2.5706 |  | + |
| 300 | fliI |  | CG1484 |  | 2.1497 |  | + | + | 0.6536 |  |  |
| 301 | Mhc |  | CG17927 |  | 2.1456 |  |  | + | -6.3266 | - | - |
| 302 | CG5853 |  | CG5853 |  | 2.0974 |  | + | + | 3.2526 |  | + |
| 303 | su(f) | Su(f) | CG17170 |  | 2.0516 |  | + | + | 2.0786 |  | + |
| 304 | CG3368 |  | CG3368 |  | 2.0233 |  | + | + | 4.1714 | + | + |
| 305 | Nrt |  | CG9704 |  | 2.0205 |  | + | + | 0.5866 |  |  |
| 306 | baf | BAF | CG7380 |  | 1.9902 |  | + | + | 0.0323 |  |  |
| 307 | CG9775 |  | CG9775 |  | 1.9898 |  | + | + | 5.2541 | + | + |
| 308 | CG10132 |  | CG10132 |  | 1.9429 |  | + | + | 5.0264 | + | + |
| 309 | CG12484 |  | CG12484 |  | 1.9131 |  | + | + | 2.5025 |  | + |
| 310 | CG13627 |  | CG13627 |  | 1.9014 |  | + | + | 1.5006 |  |  |
| 311 | Cbp80 | CBP80 | CG7035 |  | 1.8987 |  | + | + | -0.5957 |  |  |
| 312 | CoVIb |  | CG14235 |  | 1.8819 |  | + | + | -2.8735 | - | - |
| 313 | CoVb |  | CG11015 |  | 1.8721 |  | + | + | -1.2269 |  |  |
| 314 | U2af50 | U2-AF 50 | CG9998 |  | 1.8476 |  | + | + | 0.6572 |  |  |
| 315 | shot |  | CG18076 |  | 1.8378 |  | + | + | -0.6509 |  |  |
| 316 | TBPH |  | CG10327 |  | 1.8147 |  |  | + | 2.9895 |  | + |
| 317 | Lam |  | CG6944 |  | 1.7988 |  | + | + | 0.0062 |  |  |
| 318 | CanA-14F |  | CG9819 |  | 1.7985 |  | + | + | 0.0208 |  |  |
| 319 | B52 | B52 | CG10851 |  | 1.7517 |  | + | + | -0.3241 |  |  |
| 320 | vig | VIG | CG4170 |  | 1.7392 |  | + | + | 2.7867 |  | + |
| 321 | SmB |  | CG5352 |  | 1.7188 |  | + | + | -1.6650 |  | - |
| 322 | CG42748 |  | CG6448 |  | 1.7112 |  | + | + | 1.9546 |  | + |
| 323 | eys |  | CG33955 |  | 1.7069 |  | + | + | -0.5793 |  |  |
| 324 | Ref1 | REF1 | CG1101 |  | 1.6969 |  | + | + | -0.5153 |  |  |
| 325 | cd |  | CG6969 |  | 1.6957 |  | + | + | 5.3906 | + | + |
| 326 | Ppn |  | CG33103 |  | 1.6921 |  | + | + | 0.8888 |  |  |
| 327 | alpha-Cat |  | CG17947 |  | 1.6866 |  | + | + | 2.9194 | + | + |
| 328 | CoVIII |  | CG7181 |  | 1.6549 |  | + | + | -0.4006 |  |  |
| 329 | dp |  | CG33196 |  | 1.6477 |  | + | + | -1.9597 |  | - |
| 330 | Gk |  | CG7995 |  | 1.6413 |  | + | + | 3.9063 |  | + |
| 331 | SF2 | SF2 | CG6987 |  | 1.6412 |  | + | + | 0.8708 |  |  |
| 332 | Syn |  | CG3985 |  | 1.6342 |  | + | + | 0.0311 |  |  |
| 333 | Fib |  | CG9888 |  | 1.6324 |  | + | + | 0.9875 |  |  |
| 334 | hts |  | CG9325 |  | 1.6316 |  | + | + | -0.3418 |  |  |
| 335 | CG8680 |  | CG8680 |  | 1.6089 |  | + | + | -1.6412 |  | - |
| 336 | Gld |  | CG1152 |  | 1.6042 |  | + | + | -1.4307 |  |  |
| 337 | CG7772 |  | CG7772 |  | 1.5729 |  |  | + | 4.8745 | + | + |
| 338 | Tm1 |  | CG4898 |  | 1.5632 |  | + | + | -0.5787 |  |  |
| 339 | CG2061 |  | CG2061 |  | 1.5606 |  |  | + | 0.7439 |  |  |
| 340 | yps | Yps | CG5654 |  | 1.5602 |  | + | + | 0.6395 |  |  |
| 341 | VGlut |  | CG9887 |  | 1.5593 |  | + | + | 0.8206 |  |  |
| 342 | mfas |  | CG3359 |  | 1.5547 |  | + | + | -0.3334 |  |  |
| 343 | Ace |  | CG17907 |  | 1.5520 |  | + | + | 0.0415 |  |  |
| 344 | Smu1 |  | CG5451 |  | 1.5504 |  | + | + | 4.4658 |  | + |
| 345 | Mpc1 |  | CG14290 |  | 1.5280 |  | + | + | 5.1051 | + | + |
| 346 | Acp36DE |  | CG7157 |  | 1.5025 |  | + | + | -2.7858 | - | - |
| 347 | shi |  | CG18102 |  | 1.4984 |  | + | + | 0.3256 |  |  |
| 348 | lark |  | CG8597 |  | 1.4929 |  | + | + | 4.1128 |  | + |
| 349 | veli |  | CG7662 |  | 1.4912 |  | + | + | -0.3004 |  |  |
| 350 | l(2)tid |  | CG5504 |  | 1.4567 |  |  | + | -0.5053 |  |  |
| 351 | Frq1 |  | CG5744 |  | 1.4491 |  | + | + | 0.4189 |  |  |
| 352 | Unc-115b |  | CG31332 |  | 1.4484 |  | + | + | -1.1455 |  |  |
| 353 | Rbp9 |  | CG3151 |  | 1.4436 |  | + | + | 0.0611 |  |  |
| 354 | TpnC41C |  | CG2981 |  | 1.4379 |  | + | + | -2.7854 | - | - |
| 355 | Rbp2 |  | CG4429 |  | 1.4282 |  | + | + | 0.4691 |  |  |
| 356 | Tsp5D |  | CG4690 |  | 1.4171 |  | + | + | 4.9113 | + | + |
| 357 | mRpL19 |  | CG8039 |  | 1.4120 |  | + | + | 3.6740 | + | + |
| 358 | alpha-Spec |  | CG1977 |  | 1.4100 |  | + | + | -0.5783 |  |  |
| 359 | ninaG |  | CG6728 |  | 1.4034 |  |  | + | -1.0894 |  |  |
| 360 | CG1234 |  | CG1234 |  | 1.4034 |  | + | + | 0.3059 |  |  |
| 361 | Myo31DF |  | CG7438 |  | 1.3959 |  | + | + | 0.9658 |  |  |
| 362 | CG34417 |  | CG34417 |  | 1.3852 |  | + | + | 7.5867 |  | + |
| 363 | sqd | hnRNP 40 | CG16901 |  | 1.3827 |  | + | + | 2.1569 |  | + |
| 364 | l(2)03709 |  | CG15081 |  | 1.3807 |  | + | + | -0.3589 |  |  |
| 365 | pUf68 |  | CG12085 |  | 1.3792 |  | + | + | 2.4026 |  |  |
| 366 | Rbp1-like | Rbp1-like | CG1987 |  | 1.3715 |  | + | + | 0.6641 |  |  |
| 367 | Rbcn-3A |  | CG3585 |  | 1.3553 |  | + | + | 3.8529 | + | + |
| 368 | endoA |  | CG14296 |  | 1.3530 |  | + | + | 1.2888 |  |  |
| 369 | CG4721 |  | CG4721 |  | 1.3501 |  | + | + | -0.7772 |  |  |
| 370 | loqs |  | CG6866 |  | 1.3497 |  | + | + | -0.2017 |  |  |
| 371 | CG13297 |  | CG13297 |  | 1.3273 |  |  | + | 0.0951 |  |  |
| 372 | Vm34Ca |  | CG9271 |  | 1.3241 |  |  | + | 3.9429 |  | + |
| 373 | CG12400 |  | CG12400 |  | 1.3201 |  | + | + | -1.5119 |  | - |
| 374 | SmD1 | SmD1 | CG10753 |  | 1.3172 |  | + | + | 2.7550 |  | + |
| 375 | Nca |  | CG7641 |  | 1.3100 |  | + | + | 2.5277 |  |  |
| 376 | kst |  | CG12008 |  | 1.3099 |  | + | + | 0.5706 |  |  |
| 377 | Pp2B-14D |  | CG9842 |  | 1.3043 |  | + | + | 0.5150 |  |  |
| 378 | CG4612 | CG4612 | CG4612 |  | 1.2901 |  | + | + | 5.2866 | + | + |
| 379 | porin | VDAC | CG6647 |  | 1.2826 |  | + | + | -0.1761 |  |  |
| 380 | CG10077 | CG10077 | CG10077 |  | 1.2821 |  |  | + | 0.2224 |  |  |
| 381 | Dap160 |  | CG1099 |  | 1.2814 |  | + | + | -0.4006 |  |  |
| 382 | CG9132 |  | CG9132 |  | 1.2785 |  | + | + | -0.7148 |  |  |
| 383 | fne |  | CG4396 |  | 1.2759 |  | + | + | 1.0897 |  |  |
| 384 | Caps |  | CG33653 |  | 1.2718 |  | + | + | 1.7673 |  |  |
| 385 | mRpL43 |  | CG5479 |  | 1.2640 |  | + | + | 1.8659 |  |  |
| 386 | ND23 |  | CG3944 |  | 1.2586 |  | + | + | -1.0511 |  |  |
| 387 | HDAC6 |  | CG6170 |  | 1.2553 |  | + | + | 5.1074 | + | + |
| 388 | Gp210 | GP210 | CG7897 |  | 1.2548 |  | + | + | 0.7846 |  |  |
| 389 | MESK2 | MESK2 | CG15669 |  | 1.2526 |  | + | + | 3.5128 | + | + |
| 390 | Swim |  | CG3074 |  | 1.2518 |  |  | + | -1.2065 |  |  |
| 391 | CG3683 |  | CG3683 |  | 1.2514 |  | + | + | -1.5292 |  | - |
| 392 | Nc73EF |  | CG11661 |  | 1.2499 |  | + | + | 1.3424 |  | + |
| 393 | Tm1 |  | CG4898 |  | 1.2489 |  | + | + | 0.8014 |  |  |
| 394 | Galphas |  | CG2835 |  | 1.2483 |  | + | + | -1.0886 |  |  |
| 395 | Tm1 |  | CG4898 |  | 1.2481 |  |  | + | 1.0439 |  |  |
| 396 | RpS14a |  | CG1524 |  | 1.2391 |  | + | + | 0.0341 |  |  |
| 397 | beta-Spec |  | CG5870 |  | 1.2383 |  | + | + | -0.5341 |  |  |
| 398 | mRpS35 |  | CG2101 |  | 1.2375 |  | + | + | 3.6069 |  | + |
| 399 | rl | ERK | CG12559 |  | 1.2246 |  |  | + | 5.8820 | + | + |
| 400 | RpL28 |  | CG12740 |  | 1.2146 |  | + | + | 0.5382 |  |  |
| 401 | trio |  | CG18214 |  | 1.2120 |  | + | + | 5.0867 | + | + |
| 402 | CG9350 |  | CG9350 |  | 1.2083 |  | + | + | 0.8463 |  |  |
| 403 | Cpr97Ea |  | CG6131 |  | 1.2046 |  | + | + | -0.6466 |  |  |
| 404 | SmD2 | SmD2 | CG1249 |  | 1.2032 |  | + | + | 1.5766 |  |  |
| 405 | CG4038 |  | CG4038 |  | 1.2029 |  | + | + | 1.8712 |  | + |
| 406 | CG40042 |  | CG40042 |  | 1.1945 |  |  | + | -0.3333 |  |  |
| 407 | CG12079 |  | CG12079 |  | 1.1902 |  | + | + | -0.7195 |  |  |
| 408 | CG6455 |  | CG6455 |  | 1.1865 |  | + | + | -0.9004 |  |  |
| 409 | CG4587 |  | CG4587 |  | 1.1863 |  | + | + | 0.9553 |  |  |
| 410 | CG8927 |  | CG8927 |  | 1.1831 |  | + | + | 0.0356 |  |  |
| 411 | ics |  | CG9031 |  | 1.1807 |  | + | + | 0.0448 |  |  |
| 412 | Nrx-1 |  | CG7050 |  | 1.1799 |  | + | + | 0.6138 |  |  |
| 413 | Vinc |  | CG3299 |  | 1.1792 |  | + | + | -1.1104 |  |  |
| 414 | CG12203 |  | CG12203 |  | 1.1646 |  |  | + | -0.7324 |  |  |
| 415 | Cka |  | CG7392 |  | 1.1626 |  |  | + | -0.0010 |  |  |
| 416 | Hcf |  | CG1710 |  | 1.1568 |  | + | + | 4.8557 | + | + |
| 417 | Cyp4g15 |  | CG11715 |  | 1.1555 |  |  | + | 2.9171 | + | + |
| 418 | CG5214 |  | CG5214 |  | 1.1528 |  | + | + | -0.6878 |  |  |
| 419 | cpx |  | CG32490 |  | 1.1525 |  | + | + | 1.4576 |  | + |
| 420 | mtd |  | CG32464 |  | 1.1483 |  | + | + | 0.5094 |  |  |
| 421 | l(1)G0230 |  | CG2968 |  | 1.1240 |  | + | + | -0.5105 |  |  |
| 422 | SmD3 | SmD3 | CG8427 |  | 1.1230 |  | + | + | 1.4911 |  |  |
| 423 | CG9297 |  | CG9297 |  | 1.1169 |  | + | + | -0.9218 |  |  |
| 424 | Tm1 |  | CG4898 |  | 1.1166 |  |  | + | 4.1448 |  | + |
| 425 | CG3214 |  | CG3214 |  | 1.1148 |  | + | + | -1.9915 |  | - |
| 426 | CG11835 |  | CG11835 |  | 1.1131 |  | + | + | 5.0671 | + | + |
| 427 | MED4 |  | CG8609 |  | 1.1102 |  |  | + | 1.1847 |  |  |
| 428 | CG4829 |  | CG4829 |  | 1.1100 |  | + | + | 1.0243 |  |  |
| 429 | CG17271 | CG17271 | CG17271 |  | 1.1089 |  | + | + | 6.0604 | + | + |
| 430 | cpa |  | CG10540 |  | 1.1089 |  | + | + | 0.1450 |  |  |
| 431 | CG5703 |  | CG5703 |  | 1.1072 |  | + | + | -1.1992 |  |  |
| 432 | futsch |  | CG34387 |  | 1.1049 |  | + | + | -1.0181 |  |  |
| 433 | CG13601 |  | CG13601 |  | 1.1038 |  | + | + | 0.7893 |  |  |
| 434 | jbug |  | CG30092 |  | 1.0945 |  |  | + | 0.8328 |  |  |
| 435 | RpS30 |  | CG15697 |  | 1.0827 |  |  | + | 1.1872 |  |  |
| 436 | CG4169 |  | CG4169 |  | 1.0733 |  | + | + | -0.1484 |  |  |
| 437 | CG9394 |  | CG9394 |  | 1.0732 |  | + | + | -0.8836 |  |  |
| 438 | Kr-h2 |  | CG9159 |  | 1.0678 |  | + | + | 0.1657 |  |  |
| 439 | CkIalpha | CK I | CG2028 |  | 1.0553 |  | + | + | 0.3326 |  |  |
| 440 | Clect27 |  | CG3244 |  | 1.0513 |  | + | + | -1.6624 |  | - |
| 441 | opa1-like |  | CG8479 |  | 1.0463 |  | + | + | 0.3598 |  |  |
| 442 | CG17471 |  | CG17471 |  | 1.0448 |  | + | + | 3.7546 |  | + |
| 443 | rtp |  | CG10233 |  | 1.0431 |  | + | + | -0.5864 |  |  |
| 444 | Hrb27C |  | CG10377 |  | 1.0411 |  | + | + | 1.2661 |  |  |
| 445 | zip |  | CG15792 |  | 1.0364 |  | + | + | 1.0044 |  |  |
| 446 | nemy |  | CG8776 |  | 1.0340 |  | + | + | -0.7855 |  |  |
| 447 | Pur-alpha |  | CG1507 |  | 1.0319 |  | + | + | 0.3776 |  |  |
| 448 | Mlc-c |  | CG3201 |  | 1.0315 |  | + | + | -0.3905 |  |  |
| 449 | l(2)37Cc |  | CG10691 |  | 1.0196 |  | + | + | -0.0038 |  |  |
| 450 | Ank2 |  | CG32377 |  | 1.0158 |  |  | + | 3.5251 |  | + |
| 451 | stnA |  | CG12500 |  | 1.0105 |  |  | + | 2.0863 |  |  |
| 452 | CG9368 |  | CG9368 |  | 1.0073 |  | + | + | 0.3884 |  |  |
| 453 | nop5 |  | CG10206 |  | 1.0016 |  |  | + | 5.7110 | + | + |
| 454 | CG15293 |  | CG15293 |  | 0.9971 |  | + | + | 0.1310 |  |  |
| 455 | ND75 |  | CG2286 |  | 0.9969 |  | + | + | -0.5242 |  |  |
| 456 | Jupiter |  | CG31363 |  | 0.9954 |  | + | + | 6.0238 | + | + |
| 457 | poe |  | CG14472 |  | 0.9622 |  | + | + | 2.8065 |  |  |
| 458 | RpL36 |  | CG7622 |  | 0.9610 |  | + | + | 0.3871 |  |  |
| 459 | CG14762 |  | CG14762 |  | 0.9483 |  | + | + | 0.3134 |  |  |
| 460 | CG31140 |  | CG31140 |  | 0.9466 |  |  | + | 1.2409 |  |  |
| 461 | me31B |  | CG4916 |  | 0.9423 |  | + | + | 2.8720 |  | + |
| 462 | CG4747 |  | CG4747 |  | 0.9228 |  | + | + | 2.3182 | + | + |
| 463 | Fbp1 |  | CG17285 |  | 0.9103 |  | + | + | -0.1608 |  |  |
| 464 | lig |  | CG8715 |  | 0.9059 |  | + | + | 1.3572 |  |  |
| 465 | Hsp27 |  | CG4466 |  | 0.9025 |  |  | + | 0.0915 |  |  |
| 466 | CG30197 |  | CG30197 |  | 0.9017 |  |  | + | 4.4276 | + | + |
| 467 | Ank2 |  | CG34416 |  | 0.8921 |  | + | + | -1.1489 |  |  |
| 468 | mRpL44 |  | CG2109 |  | 0.8915 |  | + | + | 4.0145 | + | + |
| 469 | RpS28b |  | CG2998 |  | 0.8902 |  |  | + | -0.2535 |  |  |
| 470 | RpL29 |  | CG10071 |  | 0.8790 |  |  | + | 3.9986 |  |  |
| 471 | nwk |  | CG4684 |  | 0.8708 |  |  | + | 0.8822 |  |  |
| 472 | ATPsyn-beta |  | CG11154 |  | 0.8688 |  | + | + | -0.3308 |  |  |
| 473 | RpL23A |  | CG7977 |  | 0.8675 |  | + | + | 2.2959 |  | + |
| 474 | mRpL37 |  | CG42632 |  | 0.8646 |  |  | + | 5.0264 | + | + |
| 475 | CG6020 |  | CG6020 |  | 0.8636 |  | + | + | -0.4819 |  |  |
| 476 | Hsp23 |  | CG4463 |  | 0.8455 |  |  | + | 0.9532 |  |  |
| 477 | slgA |  | CG1417 |  | 0.8417 |  | + | + | 0.2236 |  |  |
| 478 | blw |  | CG3612 |  | 0.8225 |  | + | + | -0.8506 |  |  |
| 479 | Hml |  | CG7002 |  | 0.8196 |  |  | + | 0.5461 |  |  |
| 480 | CG8888 |  | CG8888 |  | 0.8158 |  |  | + | -0.3963 |  |  |
| 481 | comt |  | CG1618 |  | 0.8007 |  |  | + | -0.1115 |  |  |
| 482 | ATPsyn-gam. |  | CG7610 |  | 0.7980 |  |  | + | -0.7718 |  |  |
| 483 | CG3731 |  | CG3731 |  | 0.7779 |  | + | + | -0.6974 |  |  |
